# New products

**DOI:** 10.1155/S1463924696000107

**Published:** 1996


Journal of Automatic Chemistry, Vol. 18, No. 3 (May-June 1996), pp. 127-130

### New products

### On-line mercury determination

P S Analytical has recently installed systems for on-line determination of mercury in liquid samples. The system consists of a stream selection manifold, a pump, a discrete sampling valve, an atomic fluorescence spectrometer (the PSA Merlin), and an IBM compatible computer with a Process Analysis software package. It is a novel approach to process analysis and incorporates many of the facilities developed for P S Analytical's well-known Merlin Plus analytical system. Discrete analytical measurements are made in a fixed or variable sequence, rather than measuring a level continuously. This overcomes many interference problems associated with complex matrices like sulphuric acid and caustic soda.

In this application four different liquid streams can be sampled sequentially. This number can be expanded to 16 for different applications. The stream selection valve chooses the stream, then the pump delivers the sample or standard to the sampling valve. A fixed volume of sample is introduced into the digestion manifold. All of the mercury species are converted to the +2 oxidation state using the bromate/bromide digestion procedure, although different chemistries can be used. To deal with particular on-site situations, chemistries have been developed for a range of sample types, particularly relating to chlor/alkali plants. After digestion the mercury is reduced to the gaseous phase with tin (II) chloride. The gaseous mercury is separated from the liquid stream in a gas/liquid separator, then delivered to the detector after passing through a Perma Pure drying tube. The detector returns the mercury level as a transient peak.

Reagent consumption has been kept deliberately low, with flows of just 0·3 ml/min. This allows the instrument to operate unattended for up to one week. Using PSA's Control and Data Management software, automatic calibration and analysis sequences can be programmed by the user to suit particular needs. For example, the instrument can be programmed to automatically calibrate, then specify a certain number of samples before analysing standards to check that the calibration is still valid. If the calibration curve is not valid, the instrument can be programmed to automatically re-calibrate and then continue the analysis. All liquid streams are flow checked. If any have stopped, the instrument can be automatically shut down and an alarm sounded. A digital-to-analogue converter can be used to connect the instrument to a process control computer to continuously provide updated information.

The PSA Merlin detects to 1 ppb with excellent reproducibility.

The PSA 10.223 has a wide application area. It is currently being used for effluent analysis, but can be used for many applications where on-line mercury determinations in liquid samples are needed. PSA expects to extend the technology and methodologies to on-line arsenic and selenium determinations. Both the instrumentation and the on-line chemistries can be tailored by PSA's own application team to provide the most efficient and cost-effective solution for each individual application.

*For further information contact Becky Webzell at P S Analytical, Arthur House, Unit 3, Crayfields Industrial Estate, Main Road, St Pauls Cray, Orpington, Kent BR5 3HP, UK. Tel.: 01689 891211; fax: 01689 896009.*

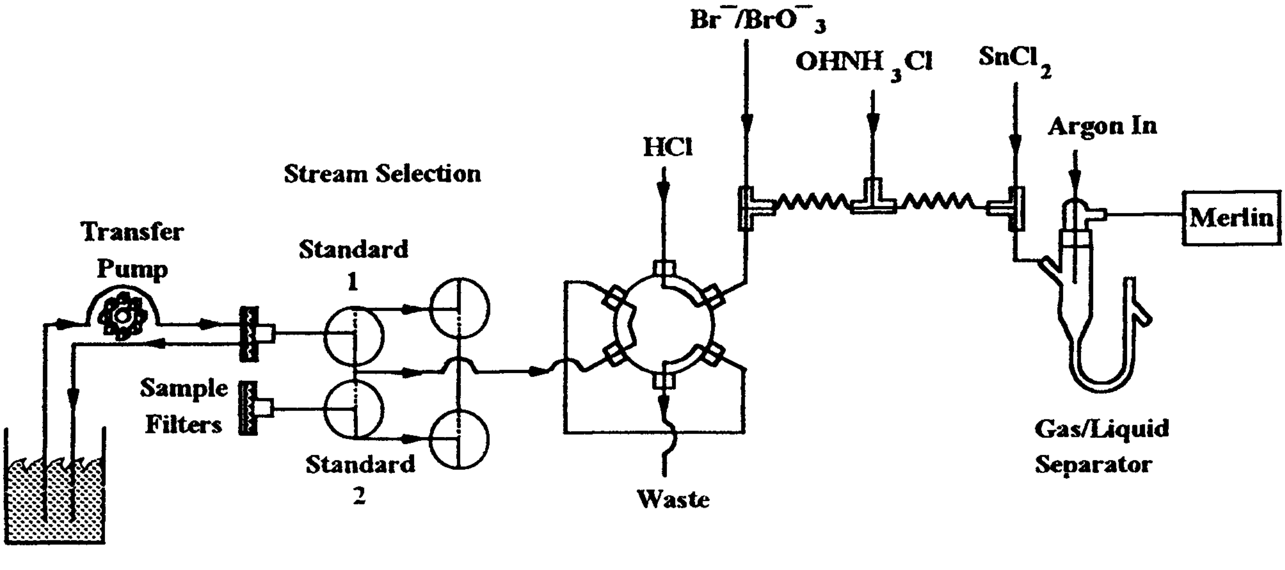

*On-line mercury determination using the PSA 10.223 system.*

0142-0453/96 $12.00 © 1996 Taylor & Francis Ltd.

### Synthetic polymer analysis

An application note from VG Organic, *The Analysis of Synthetic Polymer Formulations by Matrix Assisted Laser Desorption Ionization Time-of-Flight Mass Spectrometry*, demonstrates the applicability of MALDI-TOF to the molecular weight determination, structural elucidation and end group characterization of synthetic polymers, including polyesters and polyethers.

Mass spectrometry has traditionally been relatively limited in its applicability to the direct characterization of polymers. However, various ionization techniques can be successfully applied to the analysis of non-polymer components such as additives, modifiers and residual monomers and obligomers, which co-occur frequently with these samples. In these cases, MS is a useful tool for the industrial polymer scientist.

Until recently, Field Desorption (FD) coupled with magnetic sector MS has proved to be the most successful technique for direct analysis of intact polymers, having been reported for some samples with molecular weights exceeding 10,000. However, it is often the case that either the polymers are not amenable or their molecular weight range is far in excess of that accessible by FDMS, resulting in the need for prior chemical or thermal treatment, and then subsequent mass spectrometric analysis characterizes only the corresponding polymer repeat units.

When coupled with a Time-of-Flight (TOF) mass spectrometer, Matrix Assisted Laser Desorption Ionization (MALDI) has, more recently, been demonstrated as bridging this gap in direct mass spectrometric polymer analysis.

*For further information contact VG Organic, Queen's Avenue, Macclesfield, Cheshire SK10 2BN, UK. Tel.: 01625 434343; fax: 01625 434335.*

### Process monitoring for metals

Thermo Jarrell Ash Corporation, a subsidiary of Thermo Optek Corporation, has released a new data sheet on Process Monitoring for Metals. The automated accessory features self-standardizing, self-auditing, multiple stream monitoring and adjustable sampling frequency. Process monitoring saves raw materials and labour costs while maximizing production efficiency and minimizing waste production. The instrument is highly automated and simple to operate.

*For further information contact Thermo Jarrell Ash, 27 Forge Parkway, Franklin, MA 02038, USA.*

### Paragon 1000 improvements

Perkin-Elmer has announced improvements to the Paragon 1000 FT-IR spectrometer: these include enhanced spectral performance, validation facilities and post-run data manipulation. There is also a 50% improvement in signal-to-noise performance, enhanced GLP and validation support, greatly improved method building and running capability and better report generation, including gridded output. And the Paragon can now run partial least-squares (PLS) or principal components analysis (PLA).

*For further information contact Perkin-Elmer Limited, Post Office Lane, Beaconsfield, Buckinghamshire HP9 1QA, UK. Tel.: 01494 679026; fax: 01494 679042.*

### Laboratory chemistry exhibition

MAC '95 the International Exhibition of Chemical, Analysis, Research, Test and Biotechnology Equipment, was held from 28 November to 1 December 1995 in Milan. Some 1400 companies from 26 different countries presented new scientific instruments. 1995's MAC coincided with the 12th RICH International Chemical Exhibition and attracted 31,364 specialist visitors.

Congress events included the 10th Advanced Technology for the Clinical Laboratory and Biotechnology, which took the theme of 'Rapid Analysis Methods in the Food Sector'. The next MAC will be held in Milan from 25 to 28 November 1997.

### AtomComp 2000 brochure

Thermo Jarrell Ash Corporation, a subsidiary of Thermo Optek Corporation, has released a new brochure for the AtomComp 2000 DC Arc Spectrograph with CID Detector. The AtomComp 2000 combines the capabilities of both a direct reader and a photographic spectrograph into one system. By providing analytical power not previously available in conventional systems, the AtomComp 2000 lowers detection limits with simultaneous background correction, and performs fast qualitative and qualitative analysis without sample digestion.

*Copies from Thermo Jarrell Ash, 27 Forge Parkway, Franklin, MA 02038, USA. Tel.: 508 520 1880, ext. 200.*

### Statistics

The British Statistics program, Unistat, has been upgraded. Unistat has a number of the features of Microsoft Excel, including cut and paste, drag and drop, Wizards and an Excel-like toolbar. Unistat can import and export Excel files and can also be used as an Excel add-in without losing any of Unistat's functionality. Statistics and graphics from Unistat can also be imported directly into formatted Microsoft Word tables.

Unistat 4.0, the upgrade, is compatible with ODBC, SQL and most popular database programs. Delimited ASCII files can be imported into Unistat, so virtually any data can be brought into Unistat for statistical analysis. The program's statistical capabilities are powerful enough for specialists, yet non-statisticians can easily use the program.

Unistat can run descriptive and distribution statistics, parametric and non-parametric tests, correlation coefficients, goodness of fit tests, regression and ANOVA, multivariate analysis and time series analysis. Over 150 2D and 3D graph types can be selected from built-in chart galleries. All allow the flexibility of on-screen annotation and editing of labels, axes, graph sizes and more. Curves are automatically redrawn in proportion when a graph is resized.

*Unistat 4.0 is available in the UK from Adept Scientific plc, 6 Business Centre West, Avenue One, Letchworth, Hertfordshire, SG6 2HB, UK. Tel: 01462 480055; fax: 01462 40213. WWW: http://www.adeptscience.co.uk/.*

### Microanalysis

Oxford Instruments Microanalysis Group, which has nearly doubled in size over the last three years, is launching the Link ISIS Series 300 microanalysis system which substantially advances the capabilities originally introduced under the Link ISIS banner. The Series 300 launch encompasses both new hardware and new software. New hardware includes the DXP50 digital processor—the heart of any microanalysis system—which is claimed to provide the fastest, most accurate results currently obtainable. Sustained rates of 50,000 cps are easily achieved, leading to quantitative analyses within a 1% relative error or clear multi-element maps obtained in 5 s. With the DXP50, even at 10,000 cps, resolution using a GEM detector is typically 120 eV.

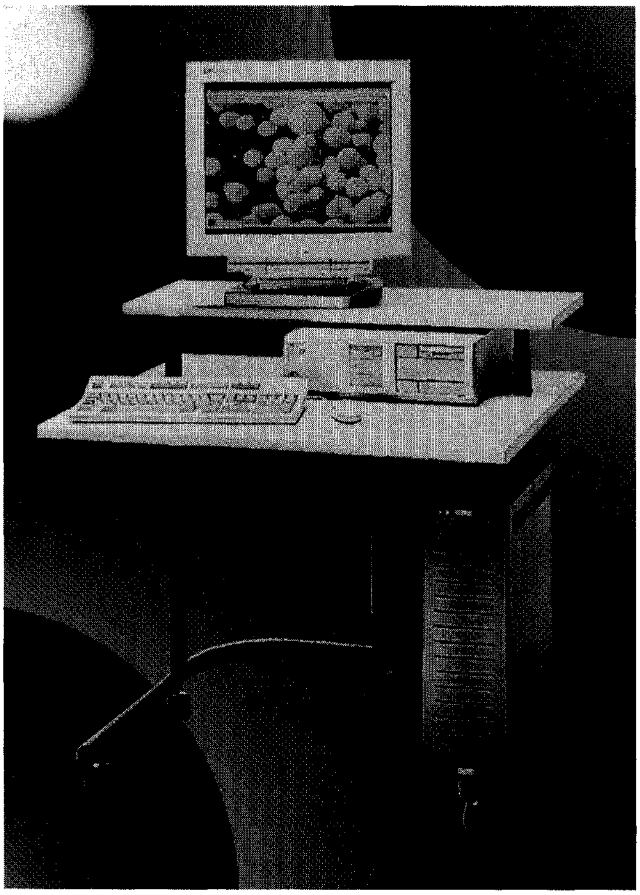

*Oxford Instruments' Link ISIS series 300 microanalysis system.*

New software facilities include 'point and quant', where a user simply has to point and click on any feature on an image to identify the elements present and display the fully corrected quantitative results. Analysis is now updated, in real time, due to the increase in precision from increased counting.

One of the features of the new Windows 95-based Link ISIS Series 300 is analysis of rough samples. This uses Oxford Instruments' peak/background technique in the system's PBQuant software.

Also available is 'Cameo', where a single mouse click acquires an image and adds colours depending on the distribution of elements present. The result of this is an easy to interpret high resolution image which combines both topographic and chemical information.

*For further information contact Oxford Instruments Microanalysis Group, Halifax Road, High Wycombe, Buckinghamshire HP12 3SE, UK. Tel.: (UK) 01494 442255; (USA) 508 369 9933; (France) 1 69 41 89 90; (Germany) 0611 764 176; (Scandinavia) 8 590 725 50; (Australia) 02 484 6108; (Japan) 03 3264 0551; (Singapore) 65 346 1556.*

### Pesticide analysis

A new application note from VG Organic describes *The Analysis of Pesticides by APCI HR-LC-MS and LC-MS-MS on an AutoSpec.* Analysts and regulatory authorities are presented with a consistent challenge when dealing with the presence of pesticides in fresh fruits and vegetables. However, a new project has provided the opportunity to both develop and improve upon a quantitative LC-MS method for pesticide detection in food. This has involved experiments with high resolution selected ion recording (HR-SIR) and multiple reaction monitoring (MRM-Q), using an AutoSpec Q mass spectrometer fitted with an atmospheric pressure chemical ionization (APCI) source. Using an artificial mixture of eight pesticides it is possible, by both HR-SIR and MRM-Q experiments, to achieve LC-MS detection limits of 1 ng or better of each when spiked at 0·05 ppm in a green bean matrix. When compared to the more usual triple quadrupole instrumentation, the high specificity of these techniques on a sector instrument can confer a significantly higher degree of confidence in the identification of these compounds.

VG Organic's new literature describes the experimental strategy required for the analysis of a mixture of eight pesticides spiked in a green bean matrix at the 0·05 ppm level by both HR-SIR and MRM-Q APCI LC-MS on an AutoSpec Q.

*Copies from Fisons Instruments, Response Handling (IAS), Queens Avenue, Macclesfield, Cheshire SK10 2BN, UK. Tel.: 01625 434343; fax: 01625 434335.*

### Refex pH and O.R.P. electrodes

R.M.S. have launched a new Division of the Company—Russell Mainstream Sensors—which is distributing Refex pH and O.R.P. electrodes from Amagruss Electrodes (Ireland) Ltd. The Refex technology is suitable for industrial and laboratory applications where the accurate measurement and control of pH and O.R.P. is important.

Refex electrodes incorporate a system in which the reference electrolyte is immobilized in an ionically conductive non-porous hard polymer that replaces the traditional liquid junction found in conventional electrodes. This overcomes the problems normally associated with conventional liquid junctions so there is no electrolyte contamination, junction clogging, drift or instability. Pressures up to 10 bar present do not affect Refex technology.

*Further information from Russell Mainstream Supply Ltd (R.M.S.), Annfield Mains House, Kettlehill, Cupar, Fife KY7 7TW, Scotland, UK. Tel.: 01337 831700.*

### Maple maths

Maple V Release 4, the latest version of the widely-used and much-acclaimed Maple V mathematical computation system, is now available. Maple V is a powerful mathematical problem-solving and visualization system; its principal strength is its *symbolic* problem-solving algorithms. Unlike conventional calculation software, which can only work with floating-point numbers, Maple V can solve problems involving formal mathematical definitions and return answers as mathematical objects.

The new Power Edition (as Release 4 is called) significantly increases the functionality of Maple V. The mathematical algorithms have been extended and now include a set of routines for dealing with piecewise-defined functions, an improved equation solver to handle inequalities and inverse trigonometric functions, and a complete package for dealing with two-dimensional geometry.

The Maple V interface has been completely redesigned to include hyperlinks, collapsible outlining, and typeset mathematics in input, output and text regions.

Maple V Release 4 is available for Windows 95, Windows NT and Windows 3.1. Versions for OS/2, Macintosh/Power Macintosh, Linux and most popular UNIX workstations platforms will be ready soon.

*Maple V is available from Adept Scientific plc, 6 Business Centre West, Avenue One, Letchworth, Hertfordshire SG6 2HB, UK. Tel.: 01462 480055; fax: 01462 480213; WWW:http://www.adeptscience.co.uk/.*

### Chrom perfect

Russell Mainstream Supply (R.M.S) Ltd, has announced a new revision of the Chrom Perfect for Windows chromatography data system. Version 3 now supports Windows NT as well as Windows 95 and Windows 3.X. Multiuser systems on networks have been greatly improved and enhanced. Version 3 allows multiple plots and reports per run and provides extensive graphical and verbal help for many operations. Graphical peak identification has also been added. Chromatograms are now selected graphically from the disk directory. Chrom Perfect continues to support data acquisition from up to 16 chromatographs per PC. Three modes of data acquisition can be combined on the same PC, including 24-bit A/D converters, HP5890 direct digital, and PE-Nelson interfaces.

*Further information from Russell Mainstream Supply Ltd (R.M.S.), Annfield Mains House, Kettlehill, Cupar, Fife KY7 7TW, Scotland, UK. Tel.: 01337 831700.*

